# Supplementary material for: Environmental Risk Factors Contributing to the Spread of Antibiotic Resistance in West Africa
Source: Microorganisms. 2025 Apr 21;13(4):951. doi: 10.3390/microorganisms13040951 (PMC12029926; doi:10.3390/microorganisms13040951)
Supplement: Supplementary file 1 [file microorganisms-13-00951-s001.zip › microorganisms-3561754-supplementary.pdf]

Environmental Risk Factors Contributing to the Spread of Antibiotic Resistance in West Africa

Adenike Adenaya [1,*], Adedapo Adedayo Adeniran [2], Chidera Linus Ugwuoke [3], Kaosara Saliu [3], Mariam Adewumi Raji [3], Amartya Rakshit [1], Mariana Ribas-Ribas [4] and Martin Könneke [1]

[1] Institute for Chemistry and Biology of the Marine Environment (ICBM), University of Oldenburg, Carl von Ossietzky Str. 9-11, 26129 Oldenburg, Germany; amartya.rakshit@uni-oldenburg.de (A.R.); martin.koenneke@uni-oldenburg.de (M.K.)
[2] Department of Pharmacognosy and Natural Medicine, Faculty of Pharmacy, University of Calabar, Calabar 540211, Nigeria; adedapo.adeniran@unical.edu.ng
[3] Department of Pharmaceutical Microbiology, Faculty of Pharmacy, University of Ibadan, Ibadan 240281, Nigeria; cugwuoke0078@stu.ui.edu.ng (C.L.U.); ksaliu0434@stu.ui.edu.ng (K.S.); mraji0375@stu.ui.edu.ng (M.A.R.)
[4] Center for Marine Sensors (ZfMarS), Institute for Chemistry and Biology of the Marine Environment (ICBM), Carl von Ossietzky University of Oldenburg,26380Wilhelmshaven, Germany; mariana.ribas.ribas@uni-oldenburg.de
* Correspondence: adenike.adenaya3@uol.de

Table S1. Antibiotic-resistant bacteria, resistance types, prevalence, genes, sources, and environmental risk factors in West African countries. "na" means not analyzed.

West African Country	Antibiotic-resistant bacteria	Common antibiotic-resistance type	Prevalence of antibiotic-resistant bacteria	Antibiotic-resistance genes	Sources	Environmental risk factors indicated	Reference
Nigeria	*Escherichia* sp.	Carbapenem resistance	> 40%	na	Hospital	Poor Hygiene	[1]
The Gambia	*Escherichia* sp. *Klebsiella* sp.	Multidrug resistance (MDR)	76% of MDR *Escherichia* sp. 24% of MDR *Klebsiella* sp.	*bla-$_{AmpH}$, bla-$_{PBP}$, CTX-M-15, bla-$_{TEM-105}$, AGlyStrB, AGlyStrA, AGlyAac3-Iia*	Hospitals	Spread within hospital settings	[2]
Nigeria	*Aeromonas* sp.	MDR	> 10% of MDR *Aeromonas* sp.	na	Aquaculture	Agriculture, aquaculture, and animal antibiotic use	[3]
Nigeria	*Salmonella* sp.	Multiple antibiotics-resistance	> 80%	na	Breeder farms	Agriculture, aquaculture, and animal antibiotic use	[4]
Nigeria	*Pseudomonas* sp. *Bacillus* sp. *Klebsiella* sp. *Leclercia* sp.	Sulfonamide-resistance	Varied depending on the antibiotics and the cell wall composition of the bacteria	*sul1, sul2*	Poultry, piggery, and cattle wastes	Agriculture, aquaculture, and animal antibiotic use	[5]
Ghana	*Escherichia* sp. *Shigella* sp. *Salmonella* sp. *Staphylococcus* sp.	Gentamycin-resistance	100% of all bacteria	na	Food	Poor Hygiene	[6]

Nigeria	*Escherichia* sp.	Extended-spectrum beta-lactamase (ESBL) and metallo beta-lactamase (MBL) production and MDR	50% of ESBL-producing *Escherichia* sp., 8% of MBL-producing *Escherichia* sp., > 90% MDR *Escherichia* sp.	Genes associated with ESBL, MBL production	Animal wastes and human samples	Poor Hygiene	[7]
Cameroon	*Staphylococcus* sp.	Methicillin-resistant *Staphylococcus aureus* (MRSA)	53% of MRSA	*mecA*	Pus, urine, blood culture, vaginal swabs, etc	Poor Hygiene	[8]
Benin Republic	*Staphylococcus* sp.	MRSA	57% of MRSA	*mecA*	Neonatology, pediatrics, maternity, operating room, and sterilization units	Spread within hospital settings	[9]
Ghana	*Bacillus* sp.<br>*Enterobacter* sp.,<br>*Acinetobacter* sp.<br>*Pseudomonas* sp.<br>*Staphylococcus* sp.	Multiple antibiotics-resistance	37% of all bacteria	na	Household dust from urban and suburban settings	Poor Hygiene	[10]
Nigeria	*Vibrio* sp.	Multiple antibiotics-resistance	> 50%	bla_{TEM}, *sul1, sul2, sul3, tetM, aphA-3, dfr, catA2, catB3*	Food	Poor Hygiene	[11]
Ghana	*Staphylococcus* sp.	Coagulase-negative Staphylococci and MDR Staphylococci	95% of antibiotic-resistant coagulase-negative *Staphylococcus* sp.<br>70% MDR coagulase-negative *Staphylococcus* sp.	Indicated through resistance markers like methicillin and penicillinase-producing strains	Urine samples from healthy individuals.	Agriculture, aquaculture, and animal antibiotic use	[12]
Cameroon	*Escherichia* sp.<br>*Klebsiella* sp.	ESBL production	64% of ESBL-producing *Escherichia* sp.<br>36% of ESBL-producing *Klebsiella* sp.	Beta-lactamase encoding genes associated with ESBL phenotype.	Infants	Spread within hospital settings	[13]
Ghana	*Aeromonas* sp.	Colistin Resistance	12% of colistin-resistant *Aeromonas* sp.	*mcr, sul1, tetE, aadA1,* bla_{OXA-2}, *ampH, imiH, qacE*	Rivers, wells, mud water, reservoirs, and irrigation ponds	Environmental pollution and waste management	[14]
Ghana	*Escherichia* sp.<br>*Klebsiella* sp.	MDR	28% of MDR *Escherichia* sp.<br>30% of MDR *Klebsiella* sp.	bla_{NDM-1}, *sull, tetO, and tetW*	Drinking water	Environmental pollution and waste management	[15]
Nigeria	*Bacillus* sp.	na	na	na	Aquaculture	Environmental pollution and waste management, Poor Hygiene	[16]
Benin Republic	*Pseudomonas* sp.<br>*Escherichia* sp.<br>*Klebsiella* sp.	ESBL production	100% of ESBL-producing *Pseudomonas sp.*,<br>33% of ESBL-producing *Escherichia* sp.,<br>75% of ESBL-producing *Klebsiella* sp.	bla_{SHV}	Community kitchens and food	Environmental pollution and waste management	[17]

Nigeria	*Klebsiella* sp.	ESBL production	> 30% of ESBL-producing *Klebsiella* sp.	bla_{TEM}, bla_{SHV}, bla_{OXA}, $bla_{CTX\text{-}M\text{-}15}$, $bla_{CTX\text{-}M\text{-}2}$, $bla_{CTX\text{-}M\text{-}9}$ bla_{VIM}, $bla_{OXA\text{-}48}$, bla_{IMP}, bla_{NDM}, bla_{KPC}	Urine, blood, wound swabs, vaginal swabs, stool	Spread within hospital settings	[18]
Nigeria	*Citrobacter* sp. *Enterobacter* sp. *Escherichia* sp. *Pseudomonas* sp. *Achromobacter* sp.	Sulfamethoxazole/trimethoprim resistance	63% of all bacteria	*sul1, sul2. dfrA1, dfrA12, and dfrA7, intI1 and intI2*	Wetlands	Environmental pollution and waste management	[19]
Nigeria	*Escherichia* sp. *Pseudomonas* sp. *Proteus* sp. *Klebsiella* sp. *Enterobacter* sp. *Serratia* sp. *Bacillus* sp. *Enterococcus* sp.	Plasmid-mediated antibiotic resistance	> 40% of all isolates, but the prevalence of Escherichia sp. resistant to all the antibiotics tested was 70%	Resistance was associated with plasmid carriage	Household water distribution tanks.	Environmental pollution and waste management, Poor Hygiene	[20]
Nigeria	*Salmonella* sp.	ESBL production and Multiple antibiotics-resistance	0% of ESBL-producing *Salmonella* sp. > 20 resistance to other antibiotics	Several resistance genes were identified through whole-genome sequencing	Human, animal environments	Poor Hygiene	[21]
Nigeria	*Escherichia* sp. *Klebsiella* sp. *Citrobacter* sp.	Carbapenem	19% of carbapenem-resistant *Enterobacteriaceae*	$bla_{TEM\text{-}1B}$, $bla_{CTX\text{-}M\text{-}15}$, $bla_{OXA\text{-}1}$, $bla_{CMY\text{-}98}$, *mcr-1, mcr-5, mcr-8*	Stool, urine, cattle, poultry, pigs, camels, hospital wastewater	Agriculture, aquaculture, and animal antibiotic use	[22]
Ghana	*Escherichia* sp.	MDR and amoxicillin-trimethoprim-resistance	51% of MDR *Escherichia* sp. 13% of amoxicillin-trimethoprim-resistant *Escherichia* sp.	na	Raw meats, ready-to-eat meats	Poor Hygiene	[23]
Ghana	*Escherichia* sp. *Klebsiella* sp. *Citrobacter* sp. *Proteus* sp.	Ampicillin and cefuroxime-resistance	100% of ampicillin and cefuroxime-resistant *Escherichia* sp. and *Klebsiella* sp.	na	Effluents from seafood processing facilities	Environmental pollution and waste management	[24]
Nigeria	*Escherichia* sp.	MDR	94% of MDR *Escherichia* sp.	*uidA*	Surface waters	Environmental pollution and waste management	[25]
Ghana	*Klebsiella* sp., *Escherichia* sp., *Enterobacter* sp.	MDR, ESBL production, carbapenemase production	50% of MDR isolates 75.6% of ESBL-producing *Klebsiella* sp. 16% of carbapenemase-producing *Klebsiella* sp.	$bla_{OXA\text{-}181}$, $bla_{CTX\text{-}M\text{-}15}$	Hospitals	Spread within hospital settings	[26]
Nigeria	*Pseudomonas* sp. *Aeromonas* sp. *Enterococcus* sp. *Bacillus* sp. *Micrococcus* sp.	MDR	High levels of MDR microplastic degraders	na	Agricultural soil	Environmental pollution and waste management	[27]

Benin Republic	*Acinetobacter* sp. *Klebsiella* sp. *Staphylococcus* sp. *Coagulase-negative staphylococci (CoNS), Escherichia* sp. *Yersinia* sp.	na	> 20% of *Acinetobacter* sp., 12% of *Klebsiella* sp., and 7% of *Coagulase-negative* staphylococci<br>Antibiotic resistance was not explicitly quantified.	na	Hospital wastewater	Environmental pollution and waste management	[28]
Nigeria	*Escherichia* sp. *Citrobacter* sp. *Enterobacter* sp.	ESBL production and MDR	16% of ESBL-producing bacterial isolates. 44% were *Escherichia* sp.<br>100% of ESBL-producing bacterial are MDR	ESBL and Beta-lactamase genes	Water	Environmental pollution and waste management	[29]
Nigeria	*Staphylococcus* sp.	MRSA	30% of MRSA	*mecA, BlaZ*<br>*tetK, tetL*<br>*ermA, ermB, ermC*<br>*dfrD, dfrK, dfrG*	Retail poultry meat markets	Agriculture, aquaculture, and animal antibiotic use	[30]
Nigeria	*Pseudomonas* sp.	Carbapenemase production	94% of carbapenemase-producing *Pseudomonas* sp.	$bla_{KPC/BIC}$, bla_{NDM}, bla_{IMP}, bla_{SPM}, bla_{AIM}, bla_{PSE-1}, *aadB, ampC, aph(3")-Ib, aph(6)-Id, tetB, tetC, tetG, floR*	Polluted urban wetlands	Environmental pollution and waste management	[31]
Ghana	*Escherichia* sp. *Klebsiella* sp. *Citrobacter* sp. *Serratia* sp. *Enterobacter* sp.	Cephalosporin-resistant ESBL productions	50% of cephalosporin-resistant isolates<br>95% of ESBL-producing *Escherichia* sp.	$bla_{CTX-M-15}$, bla_{DHA-1}, bla_{CMY-2}, bla_{NDM-1}, *CTX-M-15, AmpC,*	Humans	Environmental pollution and waste management	[32]
Ghana	*Escherichia* sp.	NDM-1-encoding plasmid	One bacterial strain harbored several antibiotic-resistance genes	bla_{NDM-1}, $bla_{CTX-M-15}$, bla_{TEM-1A}, *aadA1, aadA2, aac(6')-Ib, sul3, dfrA12, cmlA1, armA,* bla_{OXA-9}, bla_{OXA-1}	Urine	Spread within hospital settings	[33]
Ghana	*Escherichia* sp.	ESBL productions	98% of ESBL-producing *Escherichia* sp.	bla_{CTX-M}, bla_{TEM}, bla_{SHV}	Rivers	Environmental pollution and waste management	[34]
Nigeria	*Escherichia* sp. *Aeromonas* sp.	ESBL productions	58% ESBL-producing *Escherichia* sp.<br>100% of *Aeromonas* sp. resistant to cefuroxime and cefuroxime	bla_{CTX-M}, bla_{TEM}, bla_{SHV}	Rivers	Environmental pollution and waste management	[35]
Ghana	*Acinetobacter* sp. *Klebsiella* sp. *Pseudomonas* sp.	ESBL production, carbapenemase production	61% ESBL-producing bacteria<br>39% carbapenemase-producing bacteria	bla_{NDM-1}, bla_{OXA-48}. $bla_{CTX-M-15}$ bla_{TEM}, bla_{SHV}, bla_{OXA-1}, $bla_{CTX-M-15}$, bla_{TEM}, bla_{OXA}	Bedpans or trays, bed handles, mattresses, pillows, bedside carts, bedside cabinet tops and drawers,	Poor Hygiene	[36]
Nigeria	*Bacillus* sp. *Cronobacter* sp. *Acinetobacter* sp. *Enterobacter* sp.	ESBL production	30% of all isolates acquired ESBL genes.	bla_{SHV}, bla_{TEM}	Hospital wastewater	Environmental pollution and waste management	[37]

	Klebsiella sp.<br>*Enterococcus* sp.						
Benin	*Escherichia* sp.	ESBL production	50% of ESBL-producing *Escherichia* sp. in hospital effluents<br>22% of ESBL-producing *Escherichia* sp. in fecal samples and 0% ESBL-producing *Escherichia* sp. in food and water	bla_{TEM}, Bla_{OXA-1}, bla_{SHV}, $bla_{CTX-M-1}$, $bla_{CTX-M-15}$, $bla_{CTX-M-9}$	Hospital effluents, fecal matter, food, drinking water	Environmental pollution and waste management	[38]
Nigeria	*Escherichia* sp.	ESBL production	38% of ESBL-producing *Escherichia* sp. among poultry workers and their animals<br>24% of ESBL-producing *Escherichia* sp. from the surrounding environment	bla_{TEM-1}, $bla_{CTX-M-15}$, $bla_{CTX-M-65}$, bla_{OXA-1}, $bla_{OXA-129}$, *tetA*, *sul1*, *sul2*, *sul3*, *dfrA*, *aadA1*, *aadA2*, *aadA5*, *qnrB1*, *qnrS1*	Poultry workers, animals, and surrounding environments	Agriculture, aquaculture, and animal antibiotic use	[39]
Nigeria	*Escherichia* sp.<br>*Enterobacter* sp.<br>*Klebsiella* sp.<br>*Salmonella* sp.<br>*Citrobacter* sp. *Proteus* sp. and *Shigella* sp.	ESBL production	24% of ESBL-producing bacterial isolates	bla_{CTX-M}	Well water	Environmental pollution and waste management	[40]
Benin Republic	*Escherichia* sp.<br>*Staphylococcus* sp.<br>*Pseudomonas* sp.	MDR	21% of MDR *Escherichia* sp.<br>36% of MDR *Staphylococcus* sp.<br>33 % of MDR *Pseudomonas* sp.	na	Frozen chicken	Environmental pollution and waste management, Poor Hygiene	[41]
Nigeria	*Aeromonas sp.*<br>*Burkholderia* sp.<br>*Pseudomonas* sp.<br>*Enterobacter* sp.<br>*Acinetobacter* sp.	MDR	69% of MDR bacterial isolates	na	Poultry-fish farms	Agriculture, aquaculture, and animal antibiotic use	[42]
Cameroon	*Klebsiella* sp.	ESBL production,	22% of ESBL-producing *Klebsiella* sp. in pigs<br>11% of ESBL-producing *Klebsiella* sp. in abattoir workers.	$bla_{CTX-M-15}$, bla_{TEM-1B}, bla_{SHV-11}, bla_{SCO-1}, *strA*, *strB*, *oqxA*, *oqxB*, *qnrB1*, *sul1*, *sul2*, *tetA*, *dfrA15*	Pigs and abattoir workers	Poor Hygiene	[43]
Ghana	*Escherichia* sp.<br>*Pseudomonas* sp.<br>*Staphylococcus* sp.	MDR	40% of MDR *Escherichia* sp.<br>63% of MDR *Pseudomonas* sp.<br>16% of MDR *Staphylococcus* sp.<br>16% of MRSA	*mecA*, bla_{CTX-M}, bla_{SHV}, bla_{TEM}	People with filariasis	Poor Hygiene	[44]
Burkina Faso	*Escherichia* sp.<br>*Klebsiella* sp.	Carbapenemase-Production	14 % of carbapenemase-producing *Escherichia* sp.<br>18 % of carbapenemase-producing *Klebsiella* sp.	bla_{NDM}, bla_{VIM}, bla_{IMP}, bla_{KPC}, bla_{OXA-48}; *and* bla_{KPC}	Raw and treated hospital wastewater	Environmental pollution and waste management	[45]

Cameroon	*Escherichia* sp. *Klebsiella* sp. *Enterobacter* sp. *Klebsiella* sp. *Citrobacter* sp. *Serratia* sp. and *Serratia* sp.	MDR	52.7% of MDR isolates	na	Animal husbandry	Environmental pollution and waste management, Poor Hygiene	[46]
Nigeria	*Escherichia* sp. *Klebsiella* sp.	ESBL and AmpC-beta-lactamase-production	4% of ESBL-producing bacterial isolates<br>1% of AmpC-beta-lactamase-producing bacterial isolate	bla_{CTX-M}, bla_{TEM}, bla_{CMY}, bla_{OXA-4}, *qnrA*, *qnrC*, *tetA*, *tetB*	Vegetables and salads in retail outlets	Agriculture, aquaculture, and animal antibiotic use	[47]
Ghana	*Klebsiella* sp.	MDR and ESBL production	Two MDR *Klebsiella* sp. with ESBL-producing genes	bla_{NDM-1}, $bla_{CTX-M-15}$, bla_{OXA-1}, bla_{SHV-11}, bla_{TEM-1D}, *qnrS1*, *gyrA-83I*, *parC-80I*, *sul2*	Burn wounds	Spread within hospital settings	[48]
Nigeria	*Salmonella* sp.	MDR	38% of MDR *Salmonella* sp.	*sul1*, *sul2*, *floR*, bla_{CTX}, bla_{TEM}, *strA*	Food	Agriculture, aquaculture, and animal antibiotic use	[49]
Ghana	*Escherichia* sp. *Aeromonas Klebsiella* sp. sp. *Vibrio* sp.	MDR and ESBL production	19% of MDR *Escherichia* sp.<br>11% of MDR *Aeromonas* sp.<br>6% of MDR *Klebsiella* sp.<br>2% of ESBL-producing bacterial isolates	na	Food	Agriculture, aquaculture, and animal antibiotic use	[50]
Nigeria	*Escherichia* sp.	MDR and extensively drug-resistant (XDR)	82% of MDR *Escherichia* sp.<br>51%) of XDR *Escherichia* sp.	$bla_{TEM-106}$, $bla_{TEM-126}$, $bla_{CTX-M-14}$, $bla_{CTX-M-55}$, *qnrS1*, *qnrB19*, *sul1*, *sul2*, *sul3*	Live bird market	Agriculture, aquaculture, and animal antibiotic use	[51]
Burkina Faso	*Klebsiella* sp. *Klebsiella* sp. *Escherichia* sp. *Citrobacter* sp. *Proteus* sp.	ESBL production, carbapenemase production	100% were ESBL producers, 10% were carbapenemase producers	bla_{CTX-M}, bla_{NDM-1}, bla_{OXA-48}	Hospital effluents	Environmental pollution and waste management	[52]
Ghana	*Acinetobacter* sp.	Sulfamethoxazole/trimethoprim resistance, Carbapenemase production	61% of Sulfamethoxazole/trimethoprim-resistant *Acinetobacter* sp.<br>11% of carbapenemase-producing *Acinetobacter* sp.	bla_{OXA-23}, bla_{OXA-58}, $bla_{OXA-420}$ bla_{OXA-51}, bla_{OXA-70}, *blaOXA-699*, *sul1*, *tet39*, *mphE*, *msrE*, *dfrA1*, *dfrA2*	Urine, sputum, wound swabs, blood, cerebrospinal fluid, semen, and high vaginal swabs	Spread within hospital settings	[53]
Ghana	*Escherichia* sp.	ESBL production, multiple antibiotic resistance	4% of ESBL-producing *Escherichia* sp.<br>> 90% of antibiotic resistance (meropenem, ampicillin, cefuroxime, ceftriaxone, cefotaxime)	bla_{CTX-M}	Lettuce, poultry manure, irrigation water, and soil	Environmental pollution and waste management	[54]
Ghana	*Serratiamarcescens* sp. *Escherichia* sp. *Edwardsiella* sp.	Multiple antibiotic-resistance	100% resistance to ampicillin and cefuroxime	bla_{TEM}, *cmIA*, *qnrS*, *tetB*, bla_{CTX-M}	Aquaculture	Agriculture, aquaculture, and animal antibiotic use	[55]

	Citrobacter sp. *Shigella* sp.						
Nigeria	*Escherichia* sp.	MDR and enterotoxigenic-resistance	23% of MDR *Escherichia* sp. in wastewater<br>2% of MDR *Escherichia* sp. in vegetables<br>26% enterotoxigenic-resistance	na	Treated wastewater, soil, and vegetables	Environmental pollution and waste management	[56]
Nigeria	*Pseudomonas* sp.	MDR	13% of MDR *Pseudomonas* sp.	na	Wound, urine, blood, and ear swabs	Spread within hospital settings	[57]
Nigeria	*Acinetobacter* sp. *Klebsiella* sp. *Proteus* sp. *Enterobacter* sp. *Bacillus* sp.	MDR	86% of MDR bacterial isolates	*sul1*, *sul2*, *intl1*	Wastewater from pharmaceutical facilities	Environmental pollution and waste management	[58]
Ghana	*Escherichia* sp., *Aeromonas* sp., *Vibrio* sp., *Klebsiella* sp., *Serratia* sp., *Pantoea* sp.	MDR and ESBL production	82% of MDR bacterial isolates<br>48% of ESBL-producing bacterial isolates	na	Lettuce	Environmental pollution and waste management	[59]
Nigeria	*Staphylococcus* sp.	MDR and MRSA	91% of MDR *Staphylococcus* sp. and 55% of MRSA	*mecA*	Hospital effluents	Environmental pollution and waste management	[60]
Nigeria	*Escherichia* sp.	MDR	24% of MDR *Escherichia* sp. in animals,<br>16% of MDR *Escherichia* sp. in food products<br>12% of MDR *Escherichia* sp. in humans	na	Animals, food products, farm workers	Agriculture, aquaculture, and animal antibiotic use	[61]
Ghana	*Klebsiella* sp. *Escherichia* sp. *Enterobacter* sp.	MDR	30% of MDR *Klebsiella* sp.<br>28% of MDR *Escherichia* sp.<br>28% of MDR of *Enterobacter* sp.	bla_{NDM-1}, *sul1*, *sul2*, *tetO*, *tetW*	Boreholes, dams, hand-dug wells, and streams	Environmental pollution and waste management, Poor Hygiene	[62]
Ghana	*Escherichia* sp., *Pseudomonas* sp., *Aeromonas* sp.	Multiple antibiotics-resistance	High resistance towards gentamicin, amoxicillin/clavulanate, imipenem, and aztreonam	na	Stream	Environmental pollution and waste management	[63]
Burkina Faso	*Escherichia* sp.	Diarrheagenic and ESBL production	27% of Diarrheagenic *Escherichia* sp.<br>21% of ESBL-producing *Escherichia* sp.	ESBL genes	Ready-to-eat chicken	Poor Hygiene	[64]
Nigeria	*Salmonella* sp. *Escherichia* sp. *Klebsiella* sp. *Shigella* sp. *Pseudomonas* sp. and *Enterobacter* sp.	MDR	77% of MDR isolates	na	Surface water	Environmental pollution and waste management	[65]

Ghana	*Escherichia* sp., *Staphylococcus* sp. *Pseudomonas* sp.	Mono-resistance and MDR	50% of mono-resistant and MDR bacterial isolates	na	Ambient air	Environmental pollution and waste management, Poor Hygiene	[66]
Ghana	*Escherichia* sp.	MDR and ESBL production	100% of MDR *Escherichia* sp. 14% of them are ESBL-producers	*bla_{TEM–1B}*, *bla_{CTX–M–15}*	Rectal and hand swabs	Spread within hospital settings	[67]
Nigeria	*Escherichia* sp.	ESBL production	81% of ESBL-producing *Escherichia* sp. in dumpsite leachate<br>75% of ESBL-producing *Escherichia* sp. in surface waters	*bla_{CTX-M}*, *bla_{TEM}*	Dumpsite leachate and surface water	Environmental pollution and waste management	[68]
Nigeria	*Escherichia* sp., *Enterobacter* sp., *Citrobacter* sp.	ESBL production	38% of ESBL-producing bacteria (35 out of 90 isolates)	*bla_{CTX-M-15}*, *bla_{TEM-1B}*, *ampC*, *bla_{OXA-1}*	Wetlands	Environmental pollution and waste management	[69]
Senegal	*Escherichia* sp.	ESBL/AmpC-beta-lactamase-production MDR	*53% of* ESBL/ AmpC-beta-lactamase-producing *Escherichia* sp.<br>68% of MDR *Escherichia* sp.	*bla_{CTX-M-1}*, *bla_{CTX-M-8}*, *bla_{CTX-M-15,}* *qnrB*, *dfrA1*, *dfrA5*, *dfrA7*, *aadA1*	Chicken farms, drinking water	Environmental pollution and waste management	[70]
Ghana	*Escherichia* sp.	MDR	*44%* of MDR *Escherichia* sp.	*bla_{TEM-1B}*, *bla_{TEM-1C}*, *bla_{CTX-M-15}*, *bla_{DHA-1}*, *bla_{OXA-1}*, *bla_{OXA-181}*, *sul1*, *sul2*, *tetA*, *tetB*, *mphA*, *qnrS1*, *catA1*	Surface water	Environmental pollution and waste management	[71]
The Gambia	*Staphylococcus* sp.	MRSA	3% of MRSA	*mecA*	Skin, soft tissue, blood	Spread within hospital settings	[72]
Ghana	*Vibrio* sp. O1	MDR	97% of MDR *Vibrio* sp. O1	na	Water samples from various sources	Environmental pollution and waste management, Poor Hygiene	[73]
Nigeria	*Escherichia* sp.	*MDR*	92.% of MDR *Escherichia* sp.	*aadA1*, *aadA2*, *aadA5*, *armA*, *aac(3)-IIa*, *aac(6)-Ib-cr*, *aph(3)-Ia*, *aph(6)-Id*, *ant(2)-Ia*, *bla_{TEM-1}*, *bla_{CTX-M-15}*, *bla_{CTX-M-65}*, *bla_{OXA-1}*, *bla_{OXA-10}*, *bla_{OXA-129}*	Poultry farms, humans and animals	Environmental pollution and waste management, Poor Hygiene	[74]
Nigeria	*Vibrio* sp.	*MDR*	97.% of MDR *Vibrio* sp.	*sul1*, *sul2*, *bla_{pse}*, *bla_{OXA,}* *ampC*, *tetA*, *tetE*, *strA*, *aacC*	Freshwater	Environmental pollution and waste management	[75]
Ghana	*Escherichia* sp.	MDR and ESBL production	> 60% *Escherichia* sp. resistance to sulfamethoxazole/trimethoprim, ciprofloxacin and cefotaxime<br>> 60% of ESBL-producing *Escherichia* sp.	*bla_{CTX-M-15}*, *bla_{CTX-M-14}*, *bla_{CTX-M-27}*	Clinical specimens	Spread within hospital settings	[76]
Benin Republic	Diverse bacterial taxa in hospital wastewater, including	ESBL production, carbapenemase production	ESBL and carbapenemase genes and 90% of mcr variants.	*bla_{GES}*, *bla_{IMP}*, *bla_{NDM}*, *blaOXA-48*, *blaOXA-58*, *bla_{VIM}*, *mcr-5*, *mcr-3.1*, *ermB*, *tetA*,	Hospital wastewater, surface waters	Environmental pollution and waste management	[77]

and Burkina Faso	*Pseudomonas* sp. and *Acinetobacter* sp.						
Niger	Gut microbiome	Macrolide resistance and Beta-lactam resistance determinants	Macrolide treatment led to the prevalence of Beta-lactam and other antibiotic groups	*erm*	Children	Spread within hospital settings	[78]
Ghana	*Klebsiella* sp.	ESBL production	64% of ESBL-producing *Klebsiella* sp. in hospital disease samples<br>60% of ESBL-producing *Klebsiella* sp. in hospital environment	$bla_{CTX-M-15}$, $bla_{CTX-M-3}$, bla_{SHV} $bla_{OXA-181}$, bla_{OXA-48}	Humans, animals, environments	Environmental pollution and waste management, Poor Hygiene	[79]

References

1. Adegoke, A.A., Ikott, W.E. and Okoh, A.I., 2022. Carbapenem resistance associated with coliuria among outpatient and hospitalised urology patients. *New Microbes and New Infections*, *48*, p.101019.
2. Bah, S.Y., Kujabi, M.A., Darboe, S., Kebbeh, N., Kebbeh, B.F., Kanteh, A., Bojang, R., Lawn, J.E., Kampmann, B., Sesay, A.K. and de Silva, T.I., 2023. Acquisition and carriage of genetically diverse multi-drug resistant gram-negative bacilli in hospitalised newborns in The Gambia. *Communications medicine*, *3*(1), p.79.
3. Adah, D.A., Saidu, L., Oniye, S.J., Adah, A.S., Daoudu, O.B. and Ola-Fadunsin, S.D., 2024. Molecular characterization and antibiotics resistance of Aeromonas species isolated from farmed African catfish Clarias gariepinus Burchell, 1822. *BMC Veterinary Research*, *20*(1), p.16.
4. Oloso, N.O., Adeyemo, I.A., van Heerden, H., Fasanmi, O.G. and Fasina, F.O., 2019. Antimicrobial drug administration and antimicrobial resistance of salmonella isolates originating from the broiler production value chain in Nigeria. *Antibiotics*, *8*(2), p.75.
5. Adekanmbi, A.O., Adejoba, A.T., Banjo, O.A. and Saki, M., 2020. Detection of sul1 and sul2 genes in sulfonamide-resistant bacteria (SRB) from sewage, aquaculture sources, animal wastes and hospital wastewater in South-West Nigeria. *Gene Reports*, *20*, p.100742.
6. Abas, R., Cobbina, S.J. and Abakari, G., 2019. Microbial quality and antibiotic sensitivity of bacterial isolates in “Tuo-Zaafi” vended in the central business district of tamale. *Food Science & Nutrition*, *7*(11), pp.3613-3621.
7. Egbule, O.S. and Ejechi, B.O., 2021. Prevalence of extended spectrum beta-lactamases (ESBLS) producing Escherichia coli and Klebsiella pneumoniae among hospitalized patients from nigeria. *Fudma Journal Of Sciences*, *5*(2), pp.584-595.
8. Mohamadou, M., Essama, S.R., Ngonde Essome, M.C., Akwah, L., Nadeem, N., Gonsu Kamga, H., Sattar, S. and Javed, S., 2022. High prevalence of Panton-Valentine leukocidin positive, multidrug resistant, Methicillin-resistant Staphylococcus aureus strains circulating among clinical setups in Adamawa and Far North regions of Cameroon. *Plos one*, *17*(7), p.e0265118.
9. Socohou, A., Sina, H., Degbey, C., Adjobimey, T., Sossou, E., Boya, B., N’tcha, C., Adoukonou-Sagbadja, H., Adjanohoun, A. and Baba-Moussa, L., 2021. Pathogenicity and Molecular Characterization of Staphylococcus aureus Strains Isolated from the Hospital Environment of CHU-Z Abomey-Calavi/Sô-Ava (Benin). *BioMed Research International*, *2021*(1), p.6637617.
10. Tsekleves, E., de Souza, D., Pickup, R., Ahorlu, C. and Darby, A., 2023. Developing home cleaning intervention through community engagement to reduce infections and antimicrobial resistance in Ghanaian homes. *Scientific Reports*, *13*(1), p.10505.

11. Igbinosa, E.O., Beshiru, A., Igbinosa, I.H., Ogofure, A.G. and Uwhuba, K.E., 2021. Prevalence and characterization of food-borne Vibrio parahaemolyticus from African salad in southern Nigeria. *Frontiers in microbiology*, *12*, p.632266.
12. Bekoe, S.O., Hane-Weijman, S., Trads, S.L., Orman, E., Opintan, J., Hansen, M., Frimodt-Møller, N. and Styrishave, B., 2022. Reservoir of antibiotic residues and resistant coagulase negative staphylococci in a healthy population in the Greater Accra region, Ghana. *Antibiotics*, *11*(1), p.119.
13. Djim-Adjim-Ngana, K., Oumar, L.A., Mbiakop, B.W., Njifon, H.L.M., Crucitti, T., Nchiwan, E.N., Yanou, N.N. and Deweerdt, L., 2020. Prevalence of extended-spectrum beta-lactamase-producing enterobacterial urinary infections and associated risk factors in small children of Garoua, Northern Cameroon. *Pan African Medical Journal*, *36*(1).
14. Mahazu, S., Prah, I., Ota, Y., Hayashi, T., Suzuki, M., Yoshida, M., Hoshino, Y., Akeda, Y., Suzuki, T., Ishino, T. and Ablordey, A.S., 2024. Colistin Resistance Mediated by Mcr-3-Related Phosphoethanolamine Transferase Genes in Aeromonas Species Isolated from Aquatic Environments in Avaga and Pakro Communities in the Eastern Region of Ghana. *Infection and Drug Resistance*, pp.3011-3023.
15. Adzitey, F., Ekli, R. and Aduah, M., 2020. Incidence and antibiotic susceptibility of Staphylococcus aureus isolated from ready-to-eat meats in the environs of Bolgatanga Municipality of Ghana. *Cogent Environmental Science*, *6*(1), p.1791463.
16. Alhaji, N.B., Maikai, B.V. and Kwaga, J.K., 2021. Antimicrobial use, residue and resistance dissemination in freshwater fish farms of north-central Nigeria: One health implications. *Food Control*, *130*, p.108238.
17. Dougnon, V., Houssou, V.M.C., Anago, E., Nanoukon, C., Mohammed, J., Agbankpe, J., Koudokpon, H., Bouraima, B., Deguenon, E., Fabiyi, K. and Hidjo, M., 2021. Assessment of the presence of resistance genes detected from the environment and selected food products in Benin. *Journal of Environmental and Public Health*, *2021*(1), p.8420590.
18. Odewale, G., Jibola-Shittu, M.Y., Ojurongbe, O., Olowe, R.A. and Olowe, O.A., 2023. Genotypic determination of Extended Spectrum β-Lactamases and carbapenemase production in clinical isolates of Klebsiella pneumoniae in Southwest Nigeria. *Infectious Disease Reports*, *15*(3), pp.339-353.
19. Adelowo, O.O., Vollmers, J., Mäusezahl, I., Kaster, A.K. and Müller, J.A., 2018. Detection of the carbapenemase gene bla VIM-5 in members of the Pseudomonas putida group isolated from polluted Nigerian wetlands. *Scientific Reports*, *8*(1), p.15116.
20. Babalola, T.F., Olowomofe, T.O., Omodara, T.R. and Ogunyemi, T.Y., 2021. Antibiotic resistance pattern and plasmid profile of bacteria isolates from household water distribution tanks in Ado-Ekiti. *Journal of Pure and Applied Microbiology*, *15*(3), pp.1697-1705.
21. Akinyemi, K.O., Fakorede, C.O., Linde, J., Methner, U., Wareth, G., Tomaso, H. and Neubauer, H., 2023. Whole genome sequencing of Salmonella enterica serovars isolated from humans, animals, and the environment in Lagos, Nigeria. *Bmc Microbiology*, *23*(1), p.164.
22. Ngbede, E.O., Adekanmbi, F., Poudel, A., Kalalah, A., Kelly, P., Yang, Y., Adamu, A.M., Daniel, S.T., Adikwu, A.A., Akwuobu, C.A. and Abba, P.O., 2021. Concurrent resistance to carbapenem and colistin among Enterobacteriaceae recovered from human and animal sources in Nigeria is associated with multiple genetic mechanisms. *Frontiers in microbiology*, *12*, p.740348.
23. Adzitey, F., Huda, N. and Shariff, A.H.M., 2021. Phenotypic antimicrobial susceptibility of Escherichia coli from raw meats, ready-to-eat meats, and their related samples in one health context. *Microorganisms*, *9*(2), p.326.
24. Agyarkwa, M.A.K., Azaglo, G.S.K., Kokofu, H.K., Appah-Sampong, E.K., Nerquaye-Tetteh, E.N., Appoh, E., Kudjawu, J., Worlanyo, E., Batong, M.F., Akumwena, A. and Labi, A.K., 2022. Surveillance of WHO priority Gram-negative pathogenic bacteria in effluents from two seafood processing facilities in Tema, Ghana, 2021 and 2022: A descriptive study. *International Journal of Environmental Research and Public Health*, *19*(17), p.10823.

25. Adekanmbi, A.O., Adeleke, O.J., Aremu, O.O. and Olaposi, A.V., 2020. Molecular characterization, antibiogram and distribution of zntA gene in zinc-resistant Escherichia coli population recovered from anthropogenically-influenced surface water sources in Nigeria. *Meta Gene*, *26*, p.100789.
26. Labi, A.K., Bjerrum, S., Enweronu-Laryea, C.C., Ayibor, P.K., Nielsen, K.L., Marvig, R.L., Newman, M.J., Andersen, L.P. and Kurtzhals, J.A., 2020, April. High carriage rates of multidrug-resistant gram-negative bacteria in neonatal intensive care units from Ghana. In *Open Forum Infectious Diseases* (Vol. 7, No. 4, p. ofaa109). US: Oxford University Press.
27. Edet, U.O., Joseph, A.P., Nwaokorie, F.O., Okoroiwu, H.U., Udofia, U.U., Ibor, O.R., Bassey, I.U., Atim, A.D., Edet, B.O., Bassey, D.E. and Nkang, A., 2022. Impact of "sachet water" microplastic on agricultural soil physicochemistry, antibiotics resistance, bacteria diversity and function. *SN Applied Sciences*, *4*(12), p.323.
28. Deguenon, E., Dougnon, V., Houssou, V.M.C., Gbotche, E., Ahoyo, R.A., Fabiyi, K., Agbankpe, J., Mousse, W., Lougbegnon, C., Klotoe, J.R. and Tchobo, F., 2022. Hospital effluents as sources of antibiotics residues, resistant bacteria and heavy metals in Benin. *SN Applied Sciences*, *4*(8), p.206.
29. Adekanmbi, A.O., Oluwaseyi, T.A. and Oyelade, A.A., 2021. Dumpsite leachate as a hotspot of multidrug resistant Enterobacteriaceae harbouring extended spectrum and AmpC β-lactamase genes; a case study of Awotan municipal solid waste dumpsite in Southwest Nigeria. *Meta Gene*, *28*, p.100853.
30. Igbinosa, E.O., Beshiru, A., Igbinosa, I.H., Ogofure, A.G., Ekundayo, T.C. and Okoh, A.I., 2023. Prevalence, multiple antibiotic resistance and virulence profile of methicillin-resistant Staphylococcus aureus (MRSA) in retail poultry meat from Edo, Nigeria. *Frontiers in Cellular and Infection Microbiology*, *13*, p.1122059.
31. Adelowo, O.O., Helbig, T., Knecht, C., Reincke, F., Mäusezahl, I. and Müller, J.A., 2018. High abundances of class 1 integrase and sulfonamide resistance genes, and characterisation of class 1 integron gene cassettes in four urban wetlands in Nigeria. *PLoS One*, *13*(11), p.e0208269.
32. Obeng-Nkrumah, N., Hansen, D.S., Awuah-Mensah, G., Blankson, N.K., Frimodt-Møller, N., Newman, M.J., Opintan, J.A. and Krogfelt, K.A., 2023. High level of colonization with third-generation cephalosporin-resistant Enterobacterales in African community settings, Ghana. *Diagnostic microbiology and infectious disease*, *106*(1), p.115918.
33. Ayibieke, A., Sato, W., Mahazu, S., Prah, I., Addow-Thompson, J., Ohashi, M., Suzuki, T., Iwanaga, S., Ablordey, A. and Saito, R., 2018. Molecular characterisation of the NDM-1-encoding plasmid p2189-NDM in an Escherichia coli ST410 clinical isolate from Ghana. *PLoS One*, *13*(12), p.e0209623.
34. Banu, R.A., Alvarez, J.M., Reid, A.J., Enbiale, W., Labi, A.K., Ansa, E.D., Annan, E.A., Akrong, M.O., Borbor, S., Adomako, L.A. and Ahmed, H., 2021. Extended spectrum beta-lactamase Escherichia coli in river waters collected from two cities in Ghana, 2018–2020. *Tropical Medicine and Infectious Disease*, *6*(2), p.105.
35. Bisi-Johnson, M.A., Adedeji, A.A., Sulaiman, A.A., Adefisoye, M.A. and Okoh, A.I., 2023. Isolation and genotypic characterization of extended-spectrum beta-lactamase-producing Escherichia coli O157: H7 and Aeromonas hydrophila from selected freshwater sources in Southwest Nigeria. *Scientific reports*, *13*(1), p.10746.
36. Acolatse, J.E.E., Portal, E.A., Boostrom, I., Akafity, G., Dakroah, M.P., Chalker, V.J., Sands, K. and Spiller, O.B., 2022. Environmental surveillance of ESBL and carbapenemase-producing gram-negative bacteria in a Ghanaian Tertiary Hospital. *Antimicrobial Resistance & Infection Control*, *11*(1), p.49.
37. Atobatele, B.O., Akinola, O.T. and Olutona, G.O., 2023. Molecular characterization and detection of multidrug-resistant gene in bacterial strains in a health care centre located in Iwo, Osun State, Nigeria. *Scientific African*, *21*, p.e01866.
38. Sintondji, K., Fabiyi, K., Hougbenou, J., Koudokpon, H., Lègba, B., Amoussou, H., Haukka, K. and Dougnon, V., 2023. Prevalence and characterization of ESBL-producing Escherichia coli in healthy pregnant women and hospital environments in Benin: an approach based on Tricycle. *Frontiers in public health*, *11*, p.1227000.
39. Aworh, M.K., Kwaga, J., Okolocha, E., Harden, L., Hull, D., Hendriksen, R.S. and Thakur, S., 2020. Extended-spectrum ß-lactamase-producing Escherichia coli among humans, chickens and poultry environments in Abuja, Nigeria. *One Health Outlook*, *2*, pp.1-11.
40. Akinola, O.T., Onyeaghasiri, F.U., Oluranti, O.O. and Elutade, O.O., 2022. Assessment of well water as a reservoir for extended-spectrum β-lactamases (ESBL) and carbapenem resistant Enterobacteriaceae from Iwo, Osun state, Nigeria. *Iranian Journal of Microbiology*, *14*(3), p.351.

41. Anihouvi, D.G.H., Koné, K.M., Anihouvi, V.B. and Mahillon, J., 2024. Sanitary quality and bacteriological antibiotic-resistance pattern of frozen raw chicken meat sold in retail market in Benin. *Journal of Agriculture and Food Research*, *15*, p.101012.
42. Adeyemi, F.M., Ojo, O.O., Badejo, A.A., Oyedara, O.O., Olaitan, J.O., Adetunji, C.O., Hefft, D.I., Ogunjobi, A.A. and Akinde, S.B., 2022. Integrated poultry-fish farming system encourages multidrug-resistant gram-negative bacteria dissemination in pond environment and fishes. *Aquaculture*, *548*, p.737558.
43. Founou, L.L., Founou, R.C., Allam, M., Ismail, A., Djoko, C.F. and Essack, S.Y., 2018. Genome sequencing of extended-spectrum β-lactamase (ESBL)-producing Klebsiella pneumoniae isolated from pigs and abattoir workers in Cameroon. *Frontiers in microbiology*, *9*, p.188.
44. Aglomasa, B.C., Adu-Asiamah, C.K., Asiedu, S.O., Kini, P., Amewu, E.K.A., Boahen, K.G., Wireko, S., Amponsah, I.K., Boakye, Y.D., Boamah, V.E. and Kwarteng, A., 2022. Multi-drug resistant bacteria isolates from lymphatic filariasis patients in the Ahanta West District, Ghana. *BMC microbiology*, *22*(1), p.245.
45. Kagambèga, A.B., Dembélé, R., Bientz, L., M'zali, F., Mayonnove, L., Mohamed, A.H., Coulibaly, H., Barro, N. and Dubois, V., 2023. Detection and characterization of Carbapenemase-Producing Escherichia coli and Klebsiella pneumoniae from Hospital effluents of Ouagadougou, Burkina Faso. *Antibiotics*, *12*(10), p.1494.
46. Abegewi, U.A., Esemu, S.N., Ndip, R.N. and Ndip, L.M., 2022. Prevalence and risk factors of coliform-associated mastitis and antibiotic resistance of coliforms from lactating dairy cows in North West Cameroon. *PLoS one*, *17*(7), p.e0268247.
47. Beshiru, A., Igbinosa, I.H., Enabulele, T.I., Ogofure, A.G., Kayode, A.J., Okoh, A.I. and Igbinosa, E.O., 2023. Biofilm and antimicrobial resistance profile of extended-spectrum β-lactamase (ESBL) and AmpC β-lactamase producing Enterobacteriaceae in vegetables and salads. *Lwt*, *182*, p.114913.
48. Ofosu-Appiah, F., Acquah, E.E., Mohammed, J., Sakyi Addo, C., Agbodzi, B., Ofosu, D.A., Myers, C.J., Mohktar, Q., Ampomah, O.W., Ablordey, A. and Amissah, N.A., 2024. Klebsiella pneumoniae ST147 harboring bla NDM-1, multidrug resistance and hypervirulence plasmids. *Microbiology Spectrum*, *12*(3), pp.e03017-23.
49. Igbinosa, E.O., Beshiru, A., Igbinosa, I.H. and Okoh, A.I., 2022. Antimicrobial resistance and genetic characterisation of Salmonella enterica from retail poultry meats in Benin City, Nigeria. *LWT*, *169*, p.114049.
50. Baah, D.A., Kotey, F.C., Dayie, N.T., Codjoe, F.S., Tetteh-Quarcoo, P.B. and Donkor, E.S., 2022. Multidrug-resistant gram-negative bacteria contaminating raw meat sold in Accra, Ghana. *Pathogens*, *11*(12), p.1517.
51. Al-Mustapha, A.I., Alada, S.A., Raufu, I.A., Lawal, A.N., Eskola, K., Brouwer, M.S., Adetunji, V. and Heikinheimo, A., 2022. Co-occurrence of antibiotic and disinfectant resistance genes in extensively drug-resistant Escherichia coli isolated from broilers in Ilorin, North Central Nigeria. *Journal of global antimicrobial resistance*, *31*, pp.337-344.
52. Muhigwa, M., Sanou, S., Kantagba, D., Ouangraoua, S., Yehouenou, C.L., Michodigni, F., Poda, A., Renggli, E.P., Bernasconi, A., Godreuil, S. and Ouedraogo, A.S., 2023. Characterization of extended-spectrum beta-lactamase and carbapenemase genes in bacteria from environment in Burkina Faso. *The Journal of Infection in Developing Countries*, *17*(12), pp.1714-1721.
53. Ayibieke, A., Kobayashi, A., Suzuki, M., Sato, W., Mahazu, S., Prah, I., Mizoguchi, M., Moriya, K., Hayashi, T., Suzuki, T. and Iwanaga, S., 2020. Prevalence and characterization of carbapenem-hydrolyzing class D β-lactamase-producing Acinetobacter isolates from Ghana. *Frontiers in Microbiology*, *11*, p.587398.
54. Anokyewaa Appau, A.A. and Ofori, L.A., 2024. Antibiotic Resistance Profile of E. coli isolates from lettuce, poultry manure, irrigation water, and soil in Kumasi, Ghana. *International Journal of Microbiology*, *2024*(1), p.6681311.
55. Agbeko, R., Aheto, D.W., Asante, D.K., Asare, N.K., Boateng, A.A. and Adinortey, C.A., 2022. Identification of molecular determinants of antibiotic resistance in some fish farms of Ghana. *Heliyon*, *8*(9).
56. Chigor, V., Ibangha, I.A., Chigor, C. and Titilawo, Y., 2020. Treated wastewater used in fresh produce irrigation in Nsukka, Southeast Nigeria is a reservoir of enterotoxigenic and multidrug-resistant Escherichia coli. Heliyon, 6(4).

57. Adejobi, A., Ojo, O., Alaka, O., Odetoyin, B. and Onipede, A., 2021. Antibiotic resistance pattern of Pseudomonas spp. from patients in a tertiary hospital in South-West Nigeria. *Germs*, *11*(2), p.238.
58. Obayiuwana, A., Ogunjobi, A., Yang, M. and Ibekwe, M., 2018. Characterization of bacterial communities and their antibiotic resistance profiles in wastewaters obtained from pharmaceutical facilities in Lagos and Ogun States, Nigeria. *International journal of environmental research and public health*, *15*(7), p.1365.
59. Quarcoo, G., Boamah Adomako, L.A., Abrahamyan, A., Armoo, S., Sylverken, A.A., Addo, M.G., Alaverdyan, S., Jessani, N.S., Harries, A.D., Ahmed, H. and Banu, R.A., 2022. What is in the salad? Escherichia coli and antibiotic resistance in lettuce irrigated with various water sources in Ghana. *International Journal of Environmental Research and Public Health*, *19*(19), p.12722.
60. Oladipo, A.O., Oladipo, O.G. and Bezuidenhout, C.C., 2019. Multi-drug resistance traits of methicillin-resistant Staphylococcus aureus and other Staphylococcal species from clinical and environmental sources. *Journal of Water and Health*, *17*(6), pp.930-943.
61. Olowe, O.A., Adefioye, O.J., Ajayeoba, T.A., Schiebel, J., Weinreich, J., Ali, A., Burdukiewicz, M., Rödiger, S. and Schierack, P., 2019. Phylogenetic grouping and biofilm formation of multidrug resistant Escherichia coli isolates from humans, animals and food products in South-West Nigeria. *Scientific African*, *6*, p.e00158.
62. Odonkor, S.T., Simpson, S.V., Morales Medina, W.R. and Fahrenfeld, N.L., 2022. Antibiotic-Resistant Bacteria and Resistance Genes in Isolates from Ghanaian Drinking Water Sources. *Journal of Environmental and Public Health*, *2022*(1), p.2850165.
63. Adomako, L.A., Yirenya-Tawiah, D., Nukpezah, D., Abrahamya, A., Labi, A.K., Grigoryan, R., Ahmed, H., Owusu-Danquah, J., Annang, T.Y., Banu, R.A. and Osei-Atweneboana, M.Y., 2021. Reduced bacterial counts from a sewage treatment plant but increased counts and antibiotic resistance in the recipient stream in Accra, Ghana—A cross-sectional study. *Tropical medicine and infectious disease*, *6*(2), p.79.
64. Somda, N.S., Bonkoungou, O.J., Zongo, C., Kagambèga, A., Bassolé, I.H., Traoré, Y., Mahillon, J., Scippo, M.L., Hounhouigan, J.D. and Savadogo, A., 2018. Safety of ready-to-eat chicken in Burkina Faso: Microbiological quality, antibiotic resistance, and virulence genes in Escherichia coli isolated from chicken samples of Ouagadougou. *Food science & nutrition*, *6*(4), pp.1077-1084.
65. Akpan, S.N., Odeniyi, O.A., Adebowale, O., Alarape, S.A. and Adeyemo, O.K., 2020. Antibiotic resistance profile of Gram-negative bacteria isolated from Lafenwa abattoir effluent and its receiving water (Ogun River) in Abeokuta, Ogun state, Nigeria. *Onderstepoort Journal of Veterinary Research*, *87*(1), pp.1-6.
66. Azaglo, G.S.K., Khogali, M., Hann, K., Pwamang, J.A., Appoh, E., Appah-Sampong, E., Agyarkwa, M.A.K., Fiati, C., Kudjawu, J., Hedidor, G.K. and Akumwena, A., 2021. Bacteria and their antibiotic resistance profiles in ambient air in Accra, Ghana, February 2020: A Cross-Sectional Study. *Tropical medicine and infectious disease*, *6*(3), p.110.
67. Asare Yeboah, E.E., Agyepong, N., Mbanga, J., Amoako, D.G., Abia, A.L.K., Ismail, A., Owusu-Ofori, A. and Essack, S.Y., 2024. Genomic characterization of multi drug resistant ESBL-producing Escherichia coli isolates from patients and patient environments in a teaching hospital in Ghana. *BMC microbiology*, *24*(1), p.250.
68. Adekanmbi, A.O., Rabiu, A.G., Ajose, D.J., Akinlabi, O.C., Bolarinwa, K.A., Farinu, E.P., Olaposi, A.V. and Adeyemi, A.O., 2024. Solid waste dumpsite leachate and contiguous surface water contain multidrug-resistant ESBL-producing Escherichia coli carrying Extended Spectrum β-Lactamase (ESBL) genes. *BMC microbiology*, *24*(1), p.308.
69. Adelowo, O.O., Ikhimiukor, O.O., Knecht, C., Vollmers, J., Bhatia, M., Kaster, A.K. and Müller, J.A., 2020. A survey of extended-spectrum beta-lactamase-producing Enterobacteriaceae in urban wetlands in southwestern Nigeria as a step towards generating prevalence maps of antimicrobial resistance. *PLoS One*, *15*(3), p.e0229451.
70. Vounba, P., Arsenault, J., Bada-Alambédji, R. and Fairbrother, J.M., 2019. Prevalence of antimicrobial resistance and potential pathogenicity, and possible spread of third generation cephalosporin resistance, in Escherichia coli isolated from healthy chicken farms in the region of Dakar, Senegal. *PLoS One*, *14*(3), p.e0214304.

71. Tettey, R., Egyir, B., Tettey, P., Arko-Mensah, J., Addo, S.O., Owusu-Nyantakyi, C., Boateng, W. and Fobil, J., 2024. Genomic analysis of multidrug-resistant Escherichia coli from Urban Environmental water sources in Accra, Ghana, Provides Insights into public health implications. *Plos one*, *19*(5), p.e0301531.
72. Darboe, S., Dobreniecki, S., Jarju, S., Jallow, M., Mohammed, N.I., Wathuo, M., Ceesay, B., Tweed, S., Basu Roy, R., Okomo, U. and Kwambana-Adams, B., 2019. Prevalence of Panton-Valentine leukocidin (PVL) and antimicrobial resistance in community-acquired clinical Staphylococcus aureus in an urban Gambian hospital: a 11-year period retrospective pilot study. *Frontiers in cellular and infection microbiology*, *9*, p.170.
73. Abana, D., Gyamfi, E., Dogbe, M., Opoku, G., Opare, D., Boateng, G. and Mosi, L., 2019. Investigating the virulence genes and antibiotic susceptibility patterns of Vibrio cholerae O1 in environmental and clinical isolates in Accra, Ghana. *BMC infectious diseases*, *19*, pp.1-10.
74. Aworh, M.K., Kwaga, J.K., Hendriksen, R.S., Okolocha, E.C. and Thakur, S., 2021. Genetic relatedness of multidrug resistant Escherichia coli isolated from humans, chickens and poultry environments. *Antimicrobial Resistance & Infection Control*, *10*, pp.1-13.
75. Adesiyan, I.M., Bisi-Johnson, M.A. and Okoh, A.I., 2022. Incidence of antibiotic resistance genotypes of Vibrio species recovered from selected freshwaters in Southwest Nigeria. *Scientific Reports*, *12*(1), p.18912.
76. Mahazu, S., Sato, W., Ayibieke, A., Prah, I., Hayashi, T., Suzuki, T., Iwanaga, S., Ablordey, A. and Saito, R., 2022. Insights and genetic features of extended-spectrum beta-lactamase producing Escherichia coli isolates from two hospitals in Ghana. *Scientific Reports*, *12*(1), p.1843.
77. Markkanen, M.A., Haukka, K., Pärnänen, K.M., Dougnon, V.T., Bonkoungou, I.J.O., Garba, Z., Tinto, H., Sarekoski, A., Karkman, A., Kantele, A. and Virta, M.P., 2023. Metagenomic analysis of the abundance and composition of antibiotic resistance genes in hospital wastewater in Benin, Burkina Faso, and Finland. *MSphere*, *8*(1), pp.e00538-22.
78. Doan, T., Worden, L., Hinterwirth, A., Arzika, A.M., Maliki, R., Abdou, A., Zhong, L., Chen, C., Cook, C., Lebas, E. and O'Brien, K.S., 2020. Macrolide and nonmacrolide resistance with mass azithromycin distribution. *New England Journal of Medicine*, *383*(20), pp.1941-1950.
79. Calland, J.K., Haukka, K., Kpordze, S.W., Brusah, A., Corbella, M., Merla, C., Samuelsen, Ø., Feil, E.J., Sassera, D., Karikari, A.B. and Saba, C.K., 2023. Population structure and antimicrobial resistance among Klebsiella isolates sampled from human, animal, and environmental sources in Ghana: a cross-sectional genomic One Health study. *The Lancet Microbe*, *4*(11), pp.e943-e952.
